# Anti-PD-1-induced high-grade hepatitis associated with corticosteroid-resistant T cells: a case report

**DOI:** 10.1007/s00262-017-2107-7

**Published:** 2017-12-30

**Authors:** Helen M. McGuire, Elena Shklovskaya, Jarem Edwards, Paul R. Trevillian, Geoffrey W. McCaughan, Patrick Bertolino, Catriona McKenzie, Ralph Gourlay, Stuart J. Gallagher, Barbara Fazekas de St. Groth, Peter Hersey

**Affiliations:** 1 Melanoma Immunology and Oncology Unit, Centenary Institute, University of Sydney, Sydney, Australia; 2 Ramaciotti Facility for Human Systems Biology, Charles Perkins Centre, University of Sydney, Sydney, Australia; 3 Department of Biomedical Sciences, Faculty of Medicine and Health Sciences, Macquarie University, North Ryde, Australia; 4 Melanoma Institute Australia, University of Sydney, Crows Nest, Australia; 5 Newcastle Transplant Unit, John Hunter Hospital, New Lambton Heights, Australia; 6 Liver Immunology Program, Centenary Institute and AW Morrow Gastroenterology and Liver Centre, Sydney, Australia; 7 Tissue Pathology and Diagnostic Oncology, Royal Prince Alfred Hospital, Camperdown, Australia; 8 Faculty of Health and Medicine, School of Medicine and Public Health, University of Newcastle, Callaghan, Australia; 9 Discipline of Pathology, School of Medical Sciences, Charles Perkins Centre, University of Sydney, Sydney, Australia; 10 Centenary Institute, Locked Bag 6, Newtown, NSW 2042 Australia

**Keywords:** Anti-PD-1 therapy, Corticosteroids, Hepatitis, T cell, Melanoma, Immune-related adverse events

## Abstract

Effective treatment or prevention of immune side effects associated with checkpoint inhibitor therapy of cancer is an important goal in this new era of immunotherapy. Hepatitis due to immunotherapy with antibodies against PD-1 is uncommon and generally of low severity. We present an unusually severe case arising in a melanoma patient after more than 6 months uncomplicated treatment with anti-PD-1 in an adjuvant setting. The hepatitis rapidly developed resistance to high-dose steroids, requiring anti-thymocyte globulin (ATG) to achieve control. Mass cytometry allowed comprehensive phenotyping of circulating lymphocytes and revealed that CD4^+^ T cells were profoundly depleted by ATG, while CD8^+^ T cells, B cells, NK cells and monocytes were relatively spared. Multiple abnormalities in CD4^+^ T cell phenotype were stably present in the patient before disease onset. These included a population of CCR4^−^CCR6^−^ effector/memory CD4^+^ T cells expressing intermediate levels of the Th1-related chemokine receptor CXCR3 and abnormally high multi-drug resistance type 1 transporter (MDR1) activity as assessed by a rhodamine 123 excretion assay. Expression of MDR1 has been implicated in steroid resistance and may have contributed to the severity and lack of a sustained steroid response in this patient. The number of CD4^+^ rhodamine 123-excreting cells was reduced > 3.5-fold after steroid and ATG treatment. This case illustrates the need to consider this form of steroid resistance in patients failing treatment with corticosteroids. It also highlights the need for both better identification of patients at risk and the development of treatments that involve more specific immune suppression.

**Electronic supplementary material:**

The online version of this article (10.1007/s00262-017-2107-7) contains supplementary material, which is available to authorized users.

## Introduction

Immunotherapy with monoclonal antibodies (mAbs) that block PD-1-dependent negative regulation of the immune response has been a major success story in the treatment of melanoma [1–5] and other malignancies, such as lung cancer [6] and Hodgkin’s disease [7]. In spite of this achievement, anti-PD-1 therapy has been associated in some patients with severe immune-related adverse events (irAEs), which often result in cessation of immunotherapy and implementation of prolonged immunosuppressive treatments [8, 9] that can further complicate patient management [10].

PD-1 (CD279) is an inhibitory molecule that increases the threshold necessary for T cell activation and effector function. Within the T cell compartment, PD-1 is expressed by a proportion of CD4^+^ and CD8^+^ T cells that have differentiated in response to T cell receptor (TCR)-dependent recognition of antigen. PD-1 can also be expressed by other immune cells including NK cells, B cells and monocytes. PD-1 is a member of the CD28 family and engages primarily with B7 family ligands PD-L1 (B7-H1, CD274) and PD-L2 (B7-DC, CD273) [11, 12]. In T cells expressing PD-1, its ligation induces binding of the phosphatases SHP1 and SHP2. Their primary target is the CD28 co-stimulator as well as the TCR signaling proteins CD3, ZAP70 and protein kinase C [13–16]. Thus ligation of PD-1 blocks phosphorylation-dependent signaling after TCR recognition of antigen. PI3K and AKT activation are also inhibited, which reduces production of cytokines such as IL-2 and IFN-gamma and the anti-apoptotic Bcl-xL protein. Blocking PD-1 therefore promotes the function of sensitized T cells and prevents T cell death.

The PD-1/PD-L pathway has an immunoregulatory role in many tissues and is involved in the pathogenesis of several naturally occurring immune inflammatory diseases [15–18]. It is therefore not surprising that inhibition of this pathway may result in a range of irAEs that affect a variety of tissues. Induction of grade 3 or 4 hepatitis (defined as transaminase levels greater than five or eight times upper limit of normal respectively) has been reported in approximately 1–2% of patients treated with monotherapy nivolumab or pembrolizumab [2, 8, 10] and usually resolves with prompt treatment with corticosteroids and/or mycophenolate to induce immune suppression. We present a patient displaying high-grade hepatitis of protracted duration and associated with rapid development of resistance to corticosteroids. Here we review the pathogenesis of hepatitis induction by anti-PD-1 and the possible role of resistance of CD4^+^ T cells to corticosteroids in treatment of this patient.

## Materials and methods

### Study subjects and samples

PBMCs were isolated from heparin-treated whole blood using Ficoll Paque Plus (GE Healthcare, Uppsala, Sweden) and cryopreserved in vapor-phase liquid nitrogen. In addition to the female case study patient, age 57, five female melanoma patients and seven female healthy controls, with median age of 67 and 57, respectively (Table 1) were included in the study. Of the comparison melanoma patients, 3 received anti-PD-1 monotherapy in an adjuvant setting, and 2 received anti-PD-1 monotherapy in solitary with stage III–IV disease.

**Table 1:** Study participant demographics

ID	Group	Age (years)	Gender	Therapy
HP*	Hepatitis patient	57	Female	Adjuvant pembrolizumab, steroids and ATG as outlined in Fig. 1
MC1	Melanoma control	67	Female	Metastatic pembrolizumab, no ATG or steroids
MC2	Melanoma control	68	Female	Metastatic pembrolizumab, no ATG or steroids
MC3*	Melanoma control	60	Female	Adjuvant pembrolizumab, no ATG or steroids
MC4	Melanoma control	60	Female	Adjuvant pembrolizumab, no ATG or steroids
MC5	Melanoma control	68	Female	Adjuvant pembrolizumab, no ATG or steroids
HC1*	Healthy control	47	Female	N/A
HC2*	Healthy control	47	Female	N/A
HC3*	Healthy control	50	Female	N/A
HC4*	Healthy control	57	Female	N/A
HC5*	Healthy control	58	Female	N/A
HC6	Healthy control	59	Female	N/A
HC7	Healthy control	60	Female	N/A

Demographic information for study participants used in rhodamine 123 efflux assay (where indicated by asterisk) and mass cytometry immunophenotyping analysis (all participants)

**Fig. 1:**
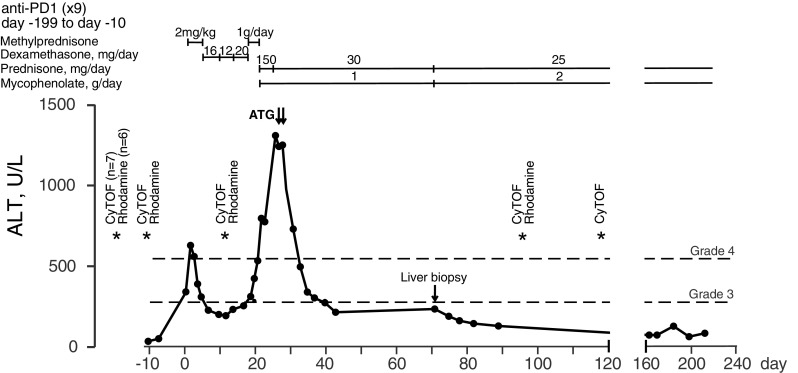
Longitudinal levels of serum alanine aminotransferase (ALT) in the anti-PD-1-treated hepatitis patient. Day 0 represents the day ALT levels exceeded Grade 3 levels. Grade 3 is defined as > 5× upper normal limit (ULN) and Grade 4 > 10× ULN. Therapeutic interventions used to control hepatitis are indicated above the graph. *ATG* anti-thymocyte globulin. Days when blood samples were processed for PBMCs and cryopreserved are indicated by asterisks, annotated by assay. CyTOF: mass cytometric analysis. Rhodamine: rhodamine 123 efflux fluorescence based analysis

### Immunohistochemistry (IHC) and multiplex immunofluorescence

Immunohistochemical and immunofluorescence staining for CD8, CD4, PD-1, and PD-L1 were carried out on 4-µm-thick sections using an Autostainer Plus (Dako-Agilent Technologies). Sections were dehydrated for 1 h at 60 °C, deparaffinized, rehydrated, and then treated with heat-induced antigen retrieval (Dako) for 20 min at 95 °C. Sections were then incubated with 3% hydrogen peroxide for 10 min, washed and then incubated with a single primary antibody [made up in Da Vinci Green Diluent solution (Biocare Medical)] for 35 min. Sections were washed and incubated with a probe antibody (Rabbit or Mouse MACH3 Probe, Biocare Medical) specific to the species of the primary antibody for 10 min, washed, and then incubated for a further 10 min with an HRP-conjugated antibody (Rabbit or Mouse MACH3 HRP, Biocare Medical) specific to the probe. For IHC staining, sections were stained with 3,3′-diaminobenzidine for 5 min and counterstained with haematoxylin for 5 min. For immunofluorescence staining sections were washed and then incubated with opal fluorophores (PerkinElmer) at a 1:50 dilution made up in tyramide signal amplification reagent (PerkinElmer). For every additional marker in the multiplex immunofluorescence assay, the process was repeated by treating the slides with an antigen retrieval step followed directly by primary antibody staining and then downstream steps. Lastly, sections were counterstained with DAPI for 3 min and then mounted (Vectashield). All Images were taken using a standard bright field microscope or a fluorescent microscope fitted with an automated quantitative pathology imaging system (Vectra) used in conjunction with Vectra 3.3 software. The following primary antibodies were used to identify CD8 expression (Cell Marque-SP16), CD4 expression (Cell Marque-SP35), PD-1 (Cell Marque-NAT205) and PD-L1 expression (Cell Signaling-E13LN).

### Mass cytometry immunophenotyping

PBMCs were thawed and washed with tissue culture medium (TCM) RPMI1640 (Thermo Fisher Scientific, Waltham, MA, USA) supplemented with 10% heat activated fetal calf serum, 2 mM l-glutamine, 25 µM 2-mercapto-ethanol, and 1000 units/L of penicillin/strep (Invitrogen). Two million PBMCs were stained for mass cytometry analyses as described [19, 20]. Briefly, PBMCs were stained with 1.25 μM cisplatin in PBS for 3 min at room temperature and quenched with TCM. Cells were incubated for 30 min at 4 °C with a 50-μL cocktail of metal-conjugated antibodies targeting surface antigens (Supplementary Table S1). Following wash with facs buffer (PBS with 5% fetal calf serum), cells were fixed and permeabilized with eBiosciences FoxP3 buffer kit (San Diego, CA, USA) according to manufacturer’s recommendations, and stained with 50 μl of intracellular antibody cocktail for 45 min at 4 °C. Cells were washed and fixed in 4% paraformaldehyde containing DNA intercalator (0.125 μM iridium-191/193; Fluidigm). After multiple washes with facs buffer and MilliQ water, cells were filtered through a 35-μm nylon mesh and diluted to 800,000 cells/mL. Cells were acquired at a rate of 200–400 cells/s using a CyTOF 2 Helios upgraded mass cytometer (Fluidigm, Toronto, Canada). Data were analyzed using the FlowJo X 10.0.7r2 software (FlowJo, LLC, Ashland, OR, USA). The *t*-stochastic neighborhood embedding (*t*-SNE) algorithm (implemented in FlowJo as a PlugIn) was utilized to perform dimensionality reduction and visualization of CD4 subpopulations across samples. A fixed number of 10,000 cells were sampled without replacement from each file and combined for analysis. The markers used for clustering were CXCR3, FOXP3, CD45RO, CCR6, CCR4, CD127, CD45RA and CCR7. The resulting *t*-SNE plots were visualized by marker expression using *t*-SNE global scaling script (https://github.com/sydneycytometry/tSNEplots).

### Flow cytometry and rhodamine 123 efflux MDR1 activity assay

Cryopreserved PBMCs were thawed and washed with TCM. Cells in cold TCM were loaded with rhodamine 123 (Sigma-Aldrich) at a final concentration of 1 µg/mL for 30 min on ice. Cells were then washed twice with cold TCM and moved to a 37 °C incubator for 2 h to allow for dye efflux. After the efflux period, cells were washed once in cold facs buffer and stained with mAbs to the following surface markers; CD45RA (HI100), CCR6 (11A9), CXCR3 (1C6/CXCR3), CD4 (RPA-T4) and CD45RO (UHCL1) from BD, CCR4 (L291H4) from Biolegend and Near IR live/dead dye from Invitrogen. Following a further wash in facs buffer, cells were fixed with 1% paraformaldehyde and acquired on a BD LSR Fortessa. Data were analyzed using FlowJo v9 software (FlowJo, LLC, Ashland, OR, USA). For some experiments 1 µM cyclosporine A (CsA; Sigma-Aldrich) or DMSO was added to cells immediately before the 37 °C incubation step.

## Results

### Description of the patient

The patient was a 57-year-old woman who had a 1.8-mm ulcerated melanoma removed from her R thigh in October 2015 followed by surgical removal of metastases in 2 of 11 lymph nodes (LNs) in the R inguinal region. She was entered into an adjuvant immunotherapy trial that compared placebo with anti-PD-1 immunotherapy given iv at 3-week intervals. After nine treatments she presented with epigastric abdominal discomfort and liver function tests revealed a pattern consistent with hepatitis. Unblinding revealed that the patient had been on treatment with anti-PD-1. Treatment of the hepatitis was initiated according to standard algorithms with methylprednisone at 2 mg/kg for 4 days (138 mg total/day) followed by oral dexamethasone at 16, 12 and 20 mg equivalent to 100, 75, 120 mg prednisone, respectively (http://clincalc.com/corticosteroids/). The course of the hepatitis as represented by serum alanine aminotransferase (ALT) levels and subsequent treatments are illustrated in Fig. 1. Changes in other liver enzymes are shown in Supplementary Figure S1. After an initial drop to below grade 3 ALT levels (< 5× upper limit of normal, ULN), ALT increased back to near grade 3 levels while on dexamethasone and did not respond to pulse dosing of methyl prednisone iv at 1 g per day for 3 days [17]. Treatment with prednisone at 150 mg and mycophenolate at 1 g/day was initiated. Due to worsening of the biochemical picture, the patient was given two i.v. doses of anti-thymocyte globulin (ATG) (Thymoglobulin, Genzyme Cambridge MA) of 100 and 50 mg, 24 h apart. Elevated ALT rapidly resolved to grade 2 levels. Low-grade elevation of ALT continued for several weeks, prompting conduct of a liver biopsy and an increase of mycophenolate dosage to 2 g/day. There was no serological evidence of hepatitis A, B or C. Low levels of smooth muscle cell antibodies (1/160) were detected but tests against mitochondrial, soluble liver and liver kidney microsomal antigens were negative. Tests against a wide panel of extractable nuclear antigens were also negative. MHC typing was HLA-A 01,02; HLA-B 08,44; HLA-C 05,07; DRB1*03,04; DPB1*03,11; DQB1*02,03; DQA1*03,05. Treatments included prophylactic anti-virals and anti-bacterials. ALT levels fluctuated between grade 1 and 2 up to day 198. ALT levels had returned to normal by day 162 relative to the ALT increase.

**Fig. 2:**
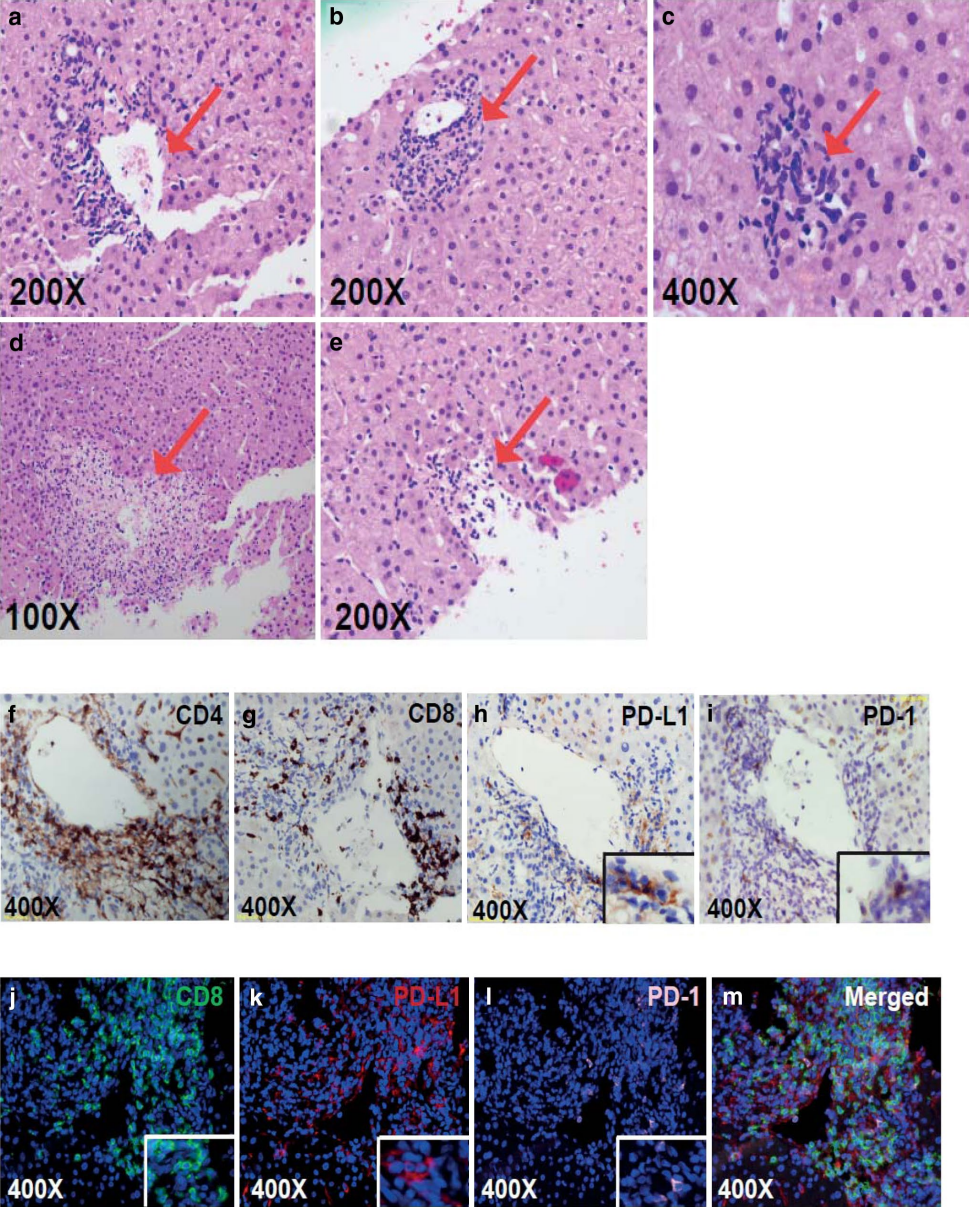
**a–e** Eosin and hematoxylin staining of the core liver biopsy. From left to right, red arrows point toward areas of the **a** portal tract, **b** endothelialitis, **c** microgranulomas, **d** the central hepatic portal vein and **e** necrosis. **f–i** Immunohistochemical staining for **f** CD4, **g** CD8, **h** PD-L1 and **i** PD-1 (t) around the central hepatic portal vein (**j–m**). Using multiplex tissue immunofluorescent staining, **j** CD8 (green), **k** PD-L1 (red), and **l** PD-1 (light pink) positive cells were identified in close proximity to one of the portal veins. The merged image **m** shows the overlap of the three markers and their proximity to each other

The H&E appearance of the liver biopsy and immunohistochemistry (IHC) are shown in Fig. 2. There was an inflammatory infiltrate around the portal tracts and central veins, with areas of focal necrosis (Fig. 2a–e) similar to other reports [10]. IHC studies in Fig. 2f, g showed that the infiltrates included both CD4^+^ and CD8^+^ T cells. As shown by the Vectra immunofluorescent images in Fig. 2j–m, PD-L1 was expressed predominantly on hepatocytes but also some of the infiltrating lymphocytes. PD-1 was expressed at low levels and was confined to infiltrating lymphocytes.

### Blood lymphocyte studies

**Fig. 3:**
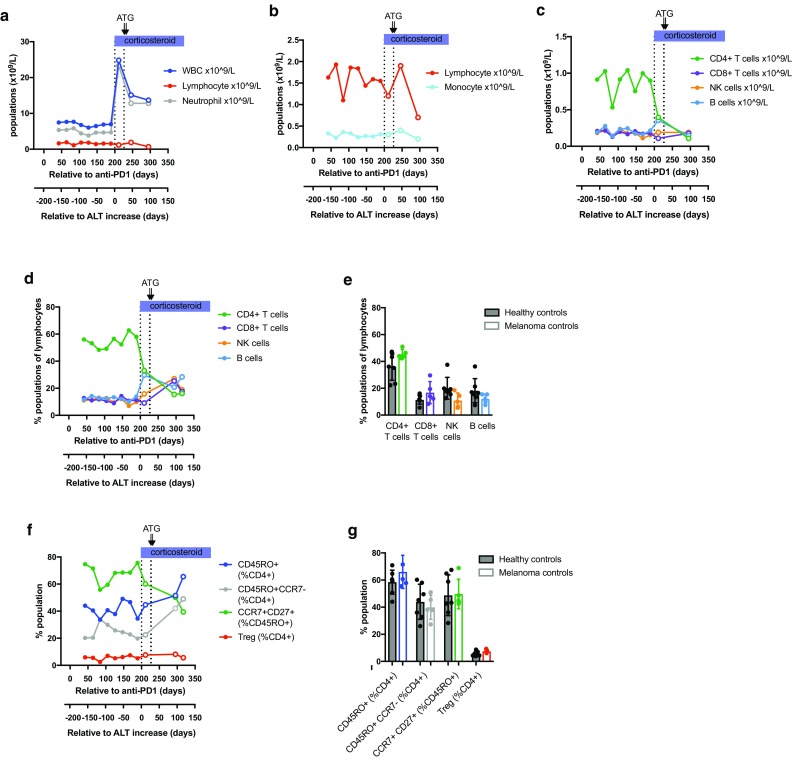
Longitudinal monitoring of peripheral blood subsets in the hepatitis patient. Density (× 10^9^/L blood) of white blood cells (WBC), lymphocytes and neutrophils are shown in **a**, with monocytes and lymphocytes shown on a narrower scale in **b. c** Density (× 10^9^/L blood) of CD4^+^ and CD8^+^ T cell, B cell and NK cell subsets, calculated from mass cytometric analysis of longitudinal samples from the hepatitis subject. **d** CD4^+^ and CD8^+^ T cell, B cell and NK cell subsets expressed as percentage of lymphocytes, calculated from mass cytometric analysis of longitudinal samples from the hepatitis subject. **e** CD4^+^ and CD8^+^ T cell, B cell and NK cell subsets expressed as percentage of lymphocytes in healthy control subjects (black filled circles, *n* = 7) and disease control patients receiving anti-PD-1 monotherapy with no irAEs (colored filled circles, *n* = 5), as described in Table 1. **f** Effector/memory CD45RO^+^, effector CD45RO^+^CCR7^−^ and Treg cells expressed as percentage of CD4^+^ T cells, and central memory CD27^+^CCR7^+^ cells expressed as percentage of CD45RO^+^CD4^+^ T cells, calculated from mass cytometric analysis of longitudinal samples from the hepatitis subject. **g** CD45RO^+^, CD45RO^+^CCR7^−^ and Treg cells expressed as percentage of CD4^+^ T cells, and CD27^+^CCR7^+^ cells expressed as percentage of CD45RO^+^CD4^+^ T cells in healthy control subjects (black filled circles, *n* = 7) and disease control patients receiving anti-PD-1 monotherapy with no irAEs (colored filled circles, *n* = 5). Therapeutic interventions used to control hepatitis are indicated above the graphs. *ATG* anti-thymocyte globulin. Open circles indicate time points at which the hepatitis patient was no longer receiving anti-PD-1 therapy

Longitudinal blood counts between 42 and 295 days after the start of anti-PD-1 treatment showed that initiation of steroid therapy on day 200 (day 1 after the onset of hepatitis) was accompanied by a dramatic rise in circulating neutrophils, as previously reported [21], with little change in lymphocyte or monocyte counts (Fig. 3a, b). Mass cytometric analysis of 11 cryopreserved PBMC samples from days 42 to 316 was performed in parallel with control PBMC samples from 5 melanoma patients on anti-PD-1 monotherapy (3 adjuvant and 2 stage III–IV disease) and 7 healthy control subjects (Table 1). In the hepatitis patient, the number of CD4^+^ T cells was reduced more than twofold in response to steroid therapy, while CD8^+^ T cells dropped by a third, and the absolute numbers of circulating NK cells and B cells increased (Fig. 3c). CD4^+^ T cell numbers declined a further fourfold in response to ATG (given on days 29 and 30 relative to ALT increase), while CD8^+^ T cells and B cells returned to pre-corticosteroid levels. Comparison of the patient’s pre-hepatitis proportions of CD4^+^ T cells, CD8^+^ T cells, NK cells and B cells with melanoma patients and healthy controls indicated that they were within the normal range (Fig. 3d, e).

Mass cytometry revealed a number of CD4^+^ T cell abnormalities that were stably present in the hepatitis patient several months before the onset of disease (Fig. 3f, g). These included a reduction in the proportion of CD45RO^+^ effector/memory CD4 T cells, most marked within the CD45RO^+^CCR7^−^ effector subset and accompanied by an abnormally high proportion of central memory phenotype CD45RO^+^CCR7^+^CD27^+^ cells These abnormalities were not corrected by steroid therapy but were reversed by ATG. In contrast, the proportion of circulating regulatory T cells (CD4^+^CD25^+^CD127^lo^FOXP3^+^) remained within normal limits throughout.

**Fig. 4:**
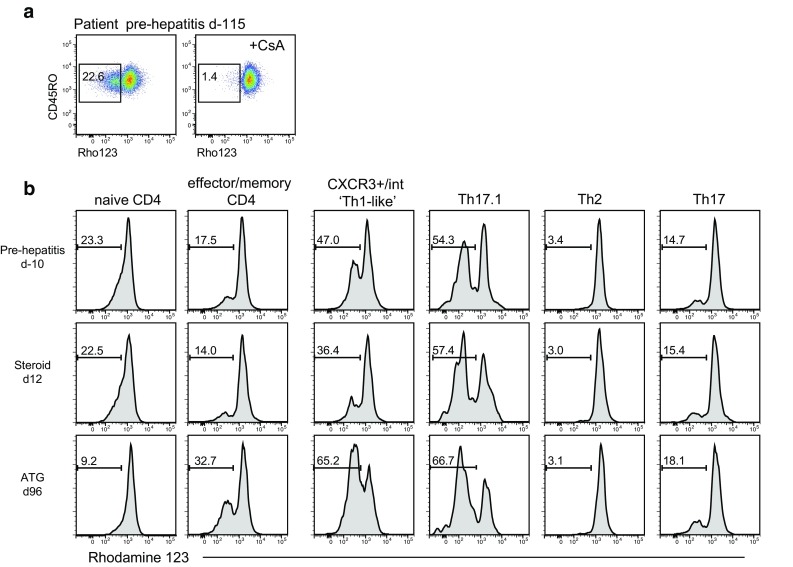
Rhodamine 123 efflux assay on PBMCs. MDR1 inhibitor cyclosporine A (CsA) was used as a control. **a** Representative plots of rhodamine exclusion by CD4^+^ T cells from the hepatitis patient. **b** Representative plots of rhodamine exclusion by CD4^+^ T cell subsets from the hepatitis patient, gated as indicated in Supplementary Figure S2. Histograms indicate rhodamine fluorescence, with frequency of the rhodamine-negative fraction indicated above the bar. Samples are annotated relative to the day of ALT increase: d-115 and d-10, pre-hepatitis; d12, after corticosteroid only; d96, after ATG therapy given at days 29 and 30

It has been reported that stable expression of MDR1 on CD4^+^ T helper (Th) 17.1 cells may render them refractory to glucocorticoid therapy [18]. We used the rhodamine 123 efflux assay as a surrogate for MDR1 function (Fig. 4a), in combination with measuring expression of chemokine receptors CCR4, CCR6 and CXCR3 on effector/memory CD45RO^+^CD4^+^ T cells to determine the distribution of CD4^+^ Th cell subsets with well-defined cytokine-producing potential [18, 22]. The gating strategy for naïve CD45RA^+^CD45RO^−^ and effector/memory CD45RA^−^CD45RO^+^ CD4^+^ T cells is illustrated in Supplementary Figure S2a. Supplementary Figure S2b, c shows the gating strategy for the CD45RA^−^CD45RO^+^ CD4^+^ Th cell subsets: Th1 (CXCR3^+^CCR4^−^CCR6^−^), Th2 (CCR4^+^CCR6^−^CXCR3^−^), Th17 (CCR4^+^CCR6^+^CXCR3^−^) and Th17.1 (CXCR3^+^CCR4^−^CCR6^+^) as applied to a healthy control and a pre-hepatitis sample. This analysis revealed that a conventional CXCR3^+^ Th1 gate excluded many cells expressing intermediate levels of CXCR3 in the hepatitis patient but not the control. A high proportion of the patient’s rhodamine-effluxing cells were within the CXCR3^int^ population and would be excluded from analysis by the application of a conventional CXCR3^+^ Th1 gate (Supplementary Figure S2d). We therefore altered the gating strategy to include both CXCR3^+^ and CXCR3^int^ cells within a ‘Th1-like’ gate (Supplementary Figure S2d). In contrast, CXCR3 was expressed at normal levels by the patient’s CCR4^−^CCR6^+^ Th17.1 cells (Supplementary Figure S2e).

We found high efflux in Th17.1 cells in all subjects (mean ± SEM 56.4 ± 1.8% in 7 pre-hepatitis samples from the patient and 46.8 ± 6.0% in samples from 5 healthy controls) (Fig. 4b). In the hepatitis patient, efflux was specifically elevated in the Th1-like subpopulation in all pre-hepatitis samples (mean ± SEM, 43.6 ± 2.3 vs 14.9 ± 2.4% in 5 controls). On d12 after the onset of hepatitis, when the patient appeared to have responded well to steroid therapy, there was a drop in the percentage of rhodamine-effluxing cells within the Th1-like population, and their absolute number was reduced 3.5-fold. By the time the last sample was taken, more than 9 weeks after ATG treatment, the proportion of rhodamine-effluxing cells had rebounded to 65.2% of Th1-like cells, although the absolute number remained low at 3.8-fold fewer than pre-hepatitis levels. Unfortunately we did not obtain a blood sample at the time that corticosteroid resistance developed, so whether it was associated with increased numbers of rhodamine-effluxing cells is not known.

Mass cytometry revealed further long-standing abnormalities in the patient’s Th1-like cells, including an increase in expression of CCR7 and CLA relative to disease controls and healthy subjects (Supplementary Figure S3). These abnormalities were unaffected or worsened after corticosteroids, but were reversed by ATG. In contrast, increased Ki67 and reduced FcrL3 and CXCR5 were unaffected by either corticosteroid or ATG treatment.

## Discussion

This case raises several questions about the occurrence of hepatitis and its severity in this patient. Grade 3–4 hepatitis is relatively rare in monotherapy with pembrolizumab or nivolumab, with rates of 0.3 and 0.7%, respectively, being recorded in large trials [2, 4]. MAbs against PD-1 are believed to act by blocking interactions of PD1 on sensitized T cells with PD-L1 on their targets, which include not only immune cells, but also by non-lymphoid tissues, such as endothelium, heart, lung, pancreas, placenta, kidney and liver [11, 23]. Expression on non-lymphoid organs is considered likely to provide regulatory feedback against autoreactive T cells that may have escaped primary tolerance mechanisms [15, 16].

It is possible that the inhibition of the PD-1/PD-L1 axis in this patient by anti-PD-1 may have allowed pre-sensitized T cells to attack the liver, although she had no history of alcohol abuse or viral infections that might have generated liver-specific autoreactive T cells. Intriguingly, the patient’s MHC class II type was DRB1 04 and DQA1*03, a genotype that is strongly associated with rheumatoid arthritis [24, 25]. This is consistent with a strong family history of rheumatoid arthritis in this patient, whose daughter, niece and sister are affected. The mechanisms whereby pre-existing autoimmune diseases or a family history of autoimmune disease contribute to irAEs after anti-PD-1 therapy remain unclear, with some patients showing no disease exacerbation while others develop new irAEs seemingly unrelated to their original autoimmune disorders [26]. It has also been speculated that the adjuvant setting may favor development of irAEs, perhaps because the absence of the tumor removes the drive for expansion of Treg populations that function to reduce the incidence of irAEs [27].

The present case is unusual, not only because the liver is rarely the target of such autoreactive responses in anti-PD-1-treated patients, but also because of the severity of the disease, which progressed in the face of treatment with high steroid doses and mycophenolate after an initial response in the first 14 days of corticosteroid treatment. Subsequent treatment with high pulse doses of steroids over 3 days also proved ineffective. Treatment with anti-thymocyte globulin (ATG) was able to arrest the process. The latter treatment was used previously in the treatment of ipilimumab-induced autoimmune hepatitis [28, 29], which usually responds to high-dose steroids [17]. Although it has been reported that ATG may act in part by induction of Treg cells [30], we found no significant change in the proportion of FOXP3^+^ Treg cells after ATG therapy. In contrast, ATG reversed the deficit in effector CD4^+^ T cells present in all the patient’s pre-hepatitis samples. The well described associations between immunodeficiency and autoimmunity [31, 32] suggest that this abnormality may have contributed to the development of hepatitis.

Evidence from the immunofluorescent and mass cytometry data in this case suggests that the effector cells causing the hepatitis were the abnormally large population of rhodamine-effluxing cells (and hence potentially steroid-resistant cells) with a ‘Th1-like’ CXCR3^int^CCR6^−^CCR4^−^ phenotype. These cells also showed heightened expression of CCR7 and CLA consistent with rapid ability to react to antigenic stimulus and enhanced accessibility to liver. These cells were evident several months prior to onset of hepatitis, and hence did not represent an adaptation to steroid therapy. The mass cytometry data showed that administration of ATG but not corticosteroids produced a stable reduction of > 3.5 fold in the number of these cells which correlated with reduction in the hepatitis. The relative deficiency in effector CD4^+^ T cells in the lead up to overt hepatitis may also have contributed to pathogenesis. It will be important to test whether these intriguing associations are present in additional patients who develop severe irAEs. This case study highlights the utility of mass cytometry to facilitate comprehensive phenotyping of circulating immune cells. This enabled us to identify and phenotype these specific immune cell subsets, which may have been missed by a more limited flow cytometry panel of markers.

The basis for resistance of lymphocytes to corticosteroids is poorly understood, but is a common problem whenever steroids are used to inhibit immune responses. It does not appear to be associated with changes in glucocorticoid receptor expression or affinity for glucocorticoids. Recent studies have drawn attention to T cell expression of MDR1, which transports steroids out of cells and can serve to select MDR1^+^ cells grown ex vivo in the presence of corticosteroids. A subset of pro-inflammatory Th17.1 cells that stably express MDR1 has been reported in patients with steroid-refractory Crohn’s disease [18]. As in the present case, activity of the MDR1 pump was shown by the ability of T cells to export rhodamine 123. Th17.1 corticosteroid-resistant T cells express CCR6, which is a receptor for CCL20, made at high levels by the liver and strongly chemotactic for lymphocytes [33]. However, the proportions of circulating Th17.1 cells with efflux of rhodamine were similar in the patient to those in controls so it is more likely the Th1 like cells were the effector cells in this case.

Although treatment of this patient followed established protocols, it may be worth considering whether assessment of MDR1 and rhodamine 123 efflux should be included in patients not responding to steroid treatment. Verapamil or cyclosporine A can inhibit MDR1 and cyclosporin A was shown to improve responses to steroids in patients with steroid unresponsive chronic obstructive airways disease [34]. Corticosteroids are relatively non-specific in their effects and ideally more specific treatments would be preferred [17, 35]. Alternative approaches used in treatment of steroid-resistant graft host disease may also be useful, e.g., adoptive transfer of mesenchymal stromal cells [36, 37], although their activity is not well established for irAEs. There also appears to be some success in treatment of acute graft versus host disease with JAK/Stat inhibitors like ruxolitinib without major side effects [38]. Treatment of inflammatory bowel disease has been a fertile area for application of therapeutic antibodies against cytokines [39] and antibodies against the IL-12p40 subunit involved in regulation of Th1 and Th17 cells appear to show promise in treatment of Crohn’s disease. These studies point to new opportunities in treatment of side effects due to checkpoint blockade, especially in patients failing corticosteroid treatments.

## Electronic supplementary material

Below is the link to the electronic supplementary material.

Supplementary material 1 (PDF 992 KB)

## Abbreviations

ALT: Alanine aminotransferase
AST: Aspartate transaminase
ATG: Anti-thymocyte globulin
CsA: Cyclosporine A
CyTOF: Cytometry by time-of-flight
GGT: Gamma-glutamyltransferase
irAEs: Immune-related adverse events
MDR1: Multi-drug resistance type 1 transporters
Pgp1: p-Glycoprotein-1
TCM: Tissue culture medium
*t*-SNE: t-Distributed stochastic neighbor embedding
ULN: Upper limit of normal
