# Supplementary material for: Anti-PD-1-induced high-grade hepatitis associated with corticosteroid-resistant T cells: a case report
Source: Cancer Immunol Immunother. 2017 Dec 30;67(4):563–73. doi: 10.1007/s00262-017-2107-7 (PMC5860100; doi:10.1007/s00262-017-2107-7)
Supplement: Supplementary file 1 — Supplementary material 1 (PDF 992 KB) [file 262_2017_2107_MOESM1_ESM.pdf]

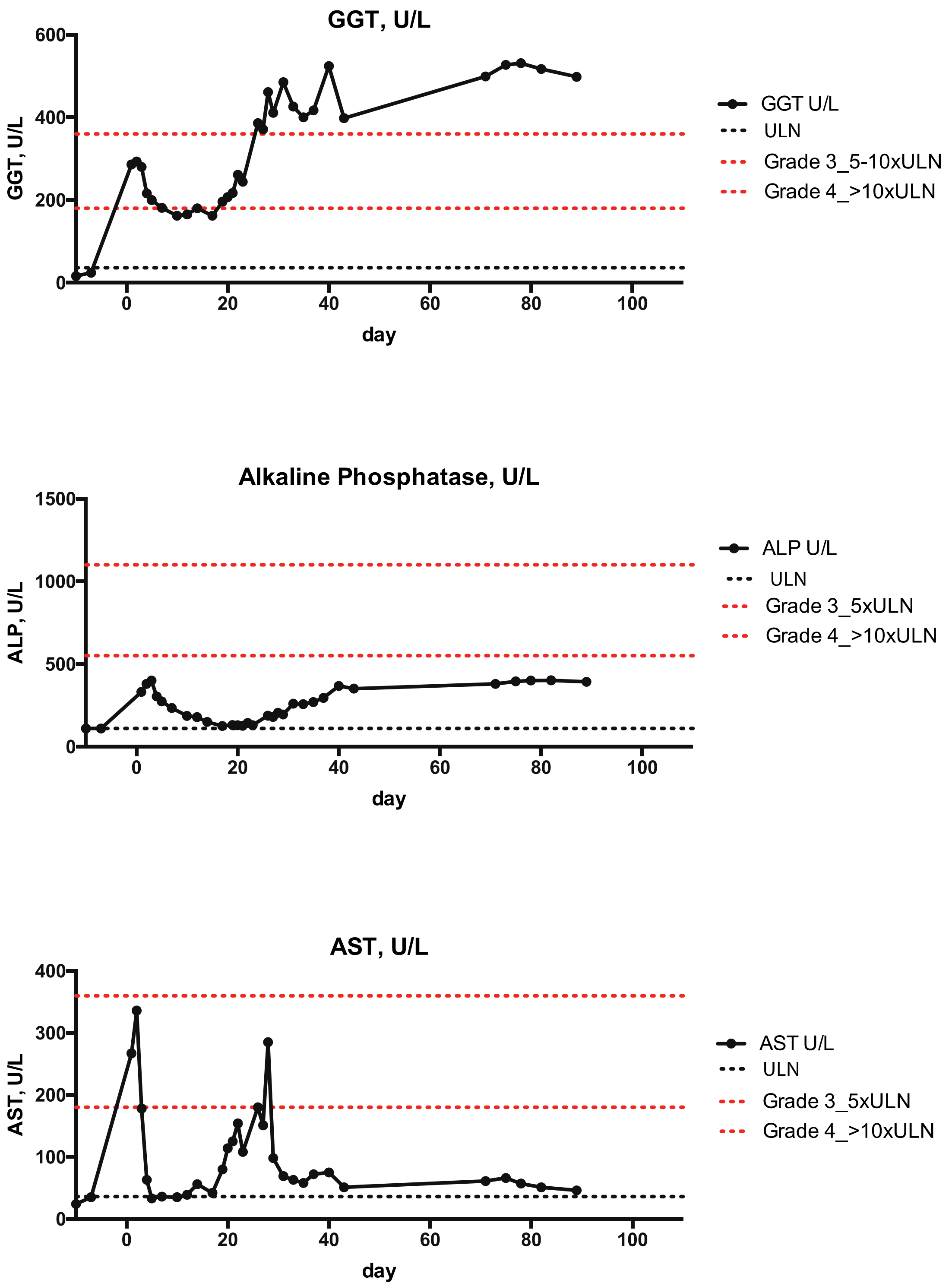

Supplementary Figure S1. Longitudinal levels of liver enzymes in the anti-PD-1 induced hepatitis patient. Day 0 represents the day on which ALT levels exceeded Grade3 levels. Levels defining grade 3 and 4 adverse reactions are shown in each graph. ULN: Upper limit of normal.

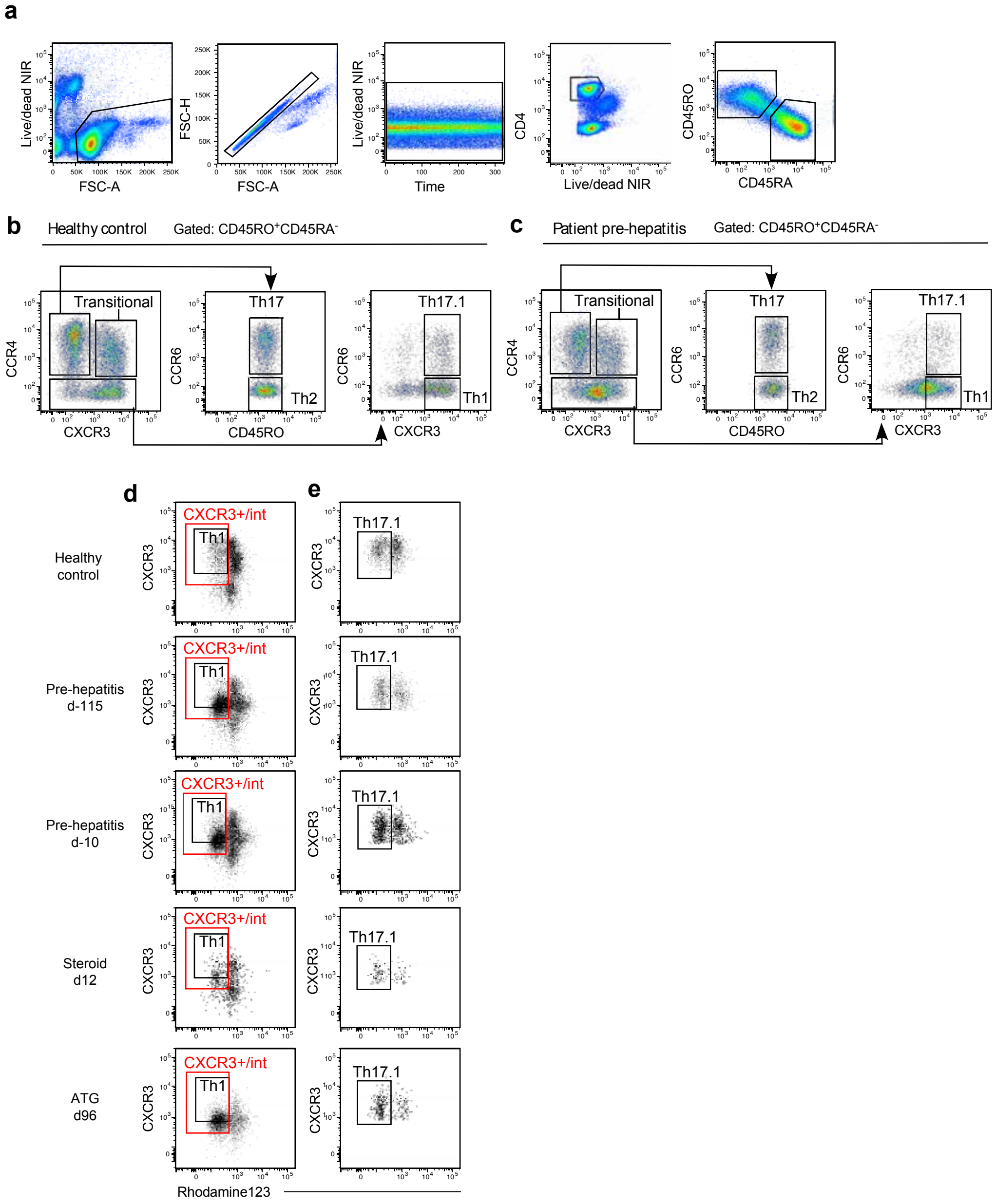

Supplementary Figure S2. (**a**) Flow cytometric gating strategy for the identification of effector/memory CD4$^+$CD45RO$^+$CD45RA$^-$ and naïve CD4$^+$CD45RO$^-$CD45RA$^+$ cells assayed for rhodamine 123 efflux as shown in Figure 4. (**b, c**) Representative plots of the gating strategy for the identification of Th subsets in normal controls (**b**) and the hepatitis patient (**c**). (**d**) Representative plots of CXCR3 expression by rhodamine effluxing cells within the CD4$^+$CD45RO$^+$CCR4$^-$CCR6$^-$ subset. The conventional CXCR3$^+$ Th1 gate is indicated in black, and the CXCR3$^{+/int}$ 'Th1-like' gate is indicated in red. (**e**) Representative plots of CXCR3 expression by rhodamine effluxing cells within the CD4$^+$CD45RO$^+$CCR4$^-$CCR6$^+$ subset. The CXCR3$^+$ Th17.1 gate is indicated in black. Samples from the hepatitis patient are annotated relative to the day of ALT increase. d-115 and d-10, pre-hepatitis; d12, after corticosteroid only; d96, after ATG therapy.

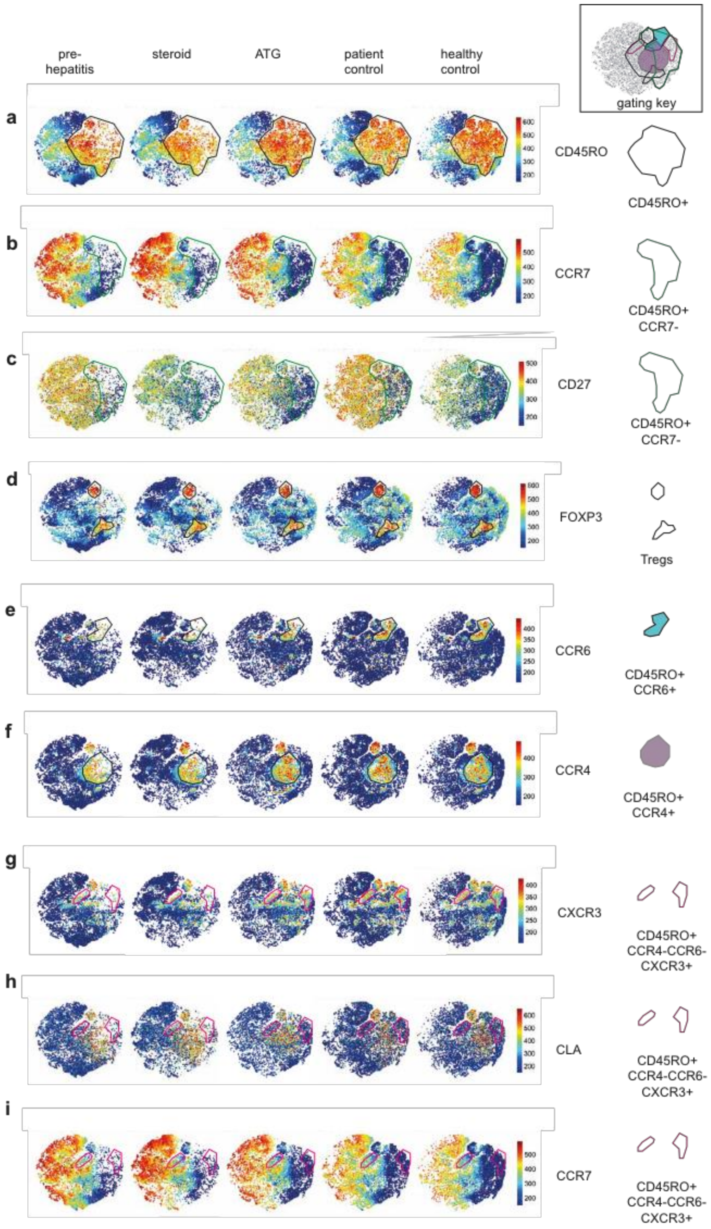

Supplementary Figure S3. Mass cytometric analysis of CD4^{+} T cells from the experiment shown in Figure 3. A concatenated file of CD4^{+} T cells from each sample was analyzed using the *t*-SNE clustering algorithm. Cells were clustered on expression of CD45RA, CD45RO, CCR7, FOXP3, CD127, CCR6, CCR4 and CXCR3. Representative plots show clustering of individual samples within the concatenated file, with individual gates shown in the gating key. (**a-i**) *t*-SNE plots colored for expression of individual markers. (**a**) CD45RO, with the gate indicating CD45RO^{+} cells, (**b**) CCR7 (gate indicating CD45RO^{+}CCR7^{-} cells), (**c**) CD27 (gate indicating CD45RO^{+}CCR7^{-} cells), (**d**) FOXP3 (gate indicating Tregs, (**e**) CCR6 (gate indicating CCR6^{+} cells within "conventional" CD45RO^{+} cells excluding Tregs), (**f**) CCR4 (gate indicating CCR4^{+} cells within "conventional" CD45RO^{+} cells), (**g**) CXCR3 (gate indicating CXCR3^{+} cells within "conventional" CD45RO^{+}CCR6^{-}CCR4^{-} cells), (**h**) CLA (gate indicating CD45RO^{+}CCR6^{-}CCR4^{-}CXCR3^{+} cells), (**i**) CCR7 (gate indicating CD45RO^{+}CCR6^{-}CCR4^{-}CXCR3^{+} cells).

Supplementary Table S1 Mass cytometry antibody panel information

Isotope/Fluorochrome	Antibody	Antibody Clone	Antibody source
Surface stain			
Pr141	CD27	M-T271	BD Biosciences*
Nd142	CD19	HIB19	Biolegend*
Nd143	CD45RA	HI100	Biolegend*
Nd145	CD4	RPA T4	BD Biosciences*
Nd146	CD8A	RPA-T8	BD Biosciences*
Sm149	CLA	HECA-452	Biolegend*
Gd154	CD196 (CCR6)	11A9	BD Biosciences*
Gd158	CD194 (CCR4)	L291H4	Biolegend*
Tb159	CD197 (CCR7)	G043H7	Biolegend*
Dy163	CD183 (CXCR3)	G025H7	Biolegend*
Er164	CD45RO	UCHL1	Biolegend*
Ho165	CD127	A019D5	Biolegend*
Tm169	CD25	2A3	BD Biosciences*
Er170	CD3	UCHT1	BD Biosciences*
Yb172	CD134 (OX40)	ACT35	BD Biosciences*
Yb174	HLA-DR	L243	Biolegend*
Intracellular stain			
Dy162	FoxP3	PCH101	eBioscience*
Live/dead marker			
Pt194/195	Cisplatin		Fluidigm

*Mass cytometry antibody conjugation protocol was carried out by the Ramaciotti Facility for Human Systems Biology, Sydney, Australia. Antibodies, without any carrier proteins were purchased from companies specified and labeled with MaxPar X8 labeling reagent kits (Fluidigm) according to the manufacturer’s instructions.
